# The TAK1→IKKβ→TPL2→MKK1/MKK2 Signaling Cascade Regulates IL-33 Expression in Cystic Fibrosis Airway Epithelial Cells Following Infection by *Pseudomonas aeruginosa*

**DOI:** 10.3389/fcell.2015.00087

**Published:** 2016-01-11

**Authors:** Raquel Farias, Simon Rousseau

**Affiliations:** Meakins-Christie Laboratories, Department of Medicine, McGill University Health Centre Research Institute, McGill UniversityMontreal, QC, Canada

**Keywords:** MAPK, ERK, Toll-like receptor, airway epithelium, *Pseudomonas aeruginosa*

## Abstract

In cystic fibrosis (CF), chronic respiratory infections result in an exaggerated and uncontrolled inflammatory response that ultimately lead to a decrease in pulmonary function. We have previously described the presence of the alarmin IL-33 in lung explants from CF patients. The signals regulating IL-33 expression in the airway epithelium following a gram-negative bacterial infection are currently unknown. Our objective was to characterize the pathways in CF airway epithelial cells (AECs) leading to an increase in IL-33 expression. We found that, in CF AECs expressing a deletion of a phenylalanine at position 508 of the gene coding for Cystic Fibrosis Transmembrane Conductance Regulator (CFTRdelF508), exposure to live *Pseudomonas aeruginosa* upregulates IL-33 via the TLR2 and TLR5 signaling pathways. This up-regulation can be partially or fully reverted by pre-incubating CFTRdelF508 AECs with a CFTR corrector (VX-809) and/or a CFTR potentiator (VX-770). Similarly, incubation with the CFTR corrector and/or the CFTR potentiator also decreased IL-8 expression in response to infection. Moreover, using different protein kinase inhibitors that target elements downstream of TLR signaling, we show that the TAK1→IKKβ→TPL2→MKK1/MKK2 pathway regulates IL-33 expression following an infection with *P. aeruginosa*. Our findings represent the first characterization of the signals regulating IL-33 expression in CF airway epithelial cells in response to a bacterial infection.

## Introduction

Cystic fibrosis (CF) is a disease caused by mutations in the gene coding for cystic fibrosis transmembrane conductance regulator (CFTR), an anion channel expressed in epithelial surfaces (Riordan, 1989). The role of CFTR is crucial for the transport of chloride (Cl^−^) and bicarbonate (${\text{HCO}}_{3}^{-}$) across cell membranes (Tabcharani et al., 1997). In the lungs, CFTR malfunction results in a hyperviscous mucus that is prone to pathogen colonization (Hoegger et al., 2014). The most prevalent pathogen in adult CF patients is the bacterium *Pseudomonas aeruginosa*. In an effort to clear the infection, airway epithelial cells release several pro-inflammatory cytokines. This inflammatory response results in neutrophil recruitment to the respiratory tract, however this response is often excessive and dysregulated, resulting in progressive and irreversible lung tissue destruction (Khan et al., 1995; Dakin et al., 2002). Bronchoalveolar lavage samples from CF patients with mild lung disease show a marked increase in neutrophil counts. This, together with increased neutrophil elastase activity has been shown to directly damage the lung (Konstan et al., 1994). Several inflammatory mediators have long been associated with chronic infection, such as interleukin (IL)-6, CXCL8 (also known as IL-8), tumor necrosis factor alpha (TNF-α) and granulocyte-macrophage colony-stimulating factor (GM-CSF) (Dakin et al., 2002; Bérubé et al., 2010). These mediators are actively released as a result of chronic exposure to bacterial components. However, tissue damage and cell death caused by an unbalanced and exaggerated immune response can also lead to the release of intracellular components that can activate receptors of the immune system. The latter molecules are known as Damage Associated Molecular Patterns (DAMPs), a group of mediators with intracellular functions that, when released following cell death, act upon Pattern Recognition Receptors (PRRs) (Piccinini and Midwood, 2010). Members of this family include DNA and RNA fragments, uric acid, high mobility group box 1 (HMGB1), S100 proteins, IL-1α and IL-33 among others (Chen and Nuñez, 2010). Increased HMGB1 and IL-1α levels have been observed in sputum from CF patients during acute exacerbations, implicating this class of mediators in the pathophysiology of the disease (Rowe et al., 2008; Colombo et al., 2011). Furthermore, increased levels of HMGB1 can predict the time to first pulmonary exacerbation, the number of future exacerbations and the time-to-lung transplantation or death (Liou et al., 2012).

IL-33 is the most recently discovered member of the IL-1 family of cytokines. Structural cells, such as endothelial cells, epithelial cells and fibroblasts, express IL-33 (Schmitz et al., 2005). IL-33 was proposed to play important functions in human diseases (Palmer and Gabay, 2011). We recently reported an increase of IL-33 in the nucleus of epithelial cells in lung explants from CF patients (Roussel et al., 2013). These findings were further supported by the discovery of the presence of IL-33 in broncho-alveolar lavage fluid of clinically stable CF patients that interestingly correlated negatively with Forced Vital Capacity, a measure of pulmonary function (Tiringer et al., 2014).

Despite the implication of IL-33 in different pathologies, the signals driving increases in IL-33 expression have not been studied in detail. It was reported that signaling through Toll-like receptors (TLRs) 2 and 4 could modulate IL-33 expression in response to infection in human monocytes (Nile et al., 2010). TLR2 and TLR4 both lead to the activation of the protein kinase TGF-beta activated kinase 1 (TAK1) in a myeloid differentiation primary response 88 (Myd88)-dependent manner. TAK1 in turn leads to the activation of Mitogen-Activated Protein Kinases (MAPKs) and nuclear factor kappa B (NFkB) signaling (Kawai and Akira, 2010). Both extracellular signal-regulated kinase (ERK)1/ERK2 and NFkB signaling require the activation of inhibitor of kappa light polypeptide gene enhancer in B-cells (IkB) kinase beta (IKKβ) by TAK1. IKKβ phosphorylates IkB leading to its degradation, allowing the release and translocation to the nucleus of NFkB (Alkalay et al., 1995). IKKβ also phosphorylates NFkB1 p105, leading to the release and activation of the protein kinase tumor progression locus-2 (TPL2), which in turn phosphorylates and activates MAPK kinase (MKK)1/MKK2, the direct upstream activators of ERK1 and ERK2 (Beinke et al., 2004). On the other hand, TAK1 also leads to the activation of JNK and p38 MAPK (Goedert, 1997; Enslen, 1998).

In this manuscript we investigated the intracellular signaling pathways responsible for increasing IL-33 expression in CFTRdelF508 AECs following an acute infection with *Pseudomonas aeruginosa*.

## Materials and methods

### Materials

5Z-7-oxozeaenol, NG25, BI605906, BIRB0796, and C1 were kindly provided by Professor Sir Philip Cohen (MRC PPU, University of Dundee, UK). PD184352 was bought from US Biological (Swampscott, MA, USA). Flagellin, Pam3CSK4, LPS, and the polyclonal antibodies against TLR2 (cat #pab-hstlr2), TLR4 (cat #pab-hstlr4), and TLR5 (cat #pab-hstlr5) were purchased from InvivoGen (San Diego, CA, USA). Antibodies against p44/42 ERK MAPK (cat #9107) and phospho-ERK Thr^202^/Tyr^204^ (cat #4370) were purchased from Cell Signaling Technology. Recombinant human IL-1β was purchased from R&D systems (Minneapolis, MN, USA). The clinical isolate of *Pseudomonas aeruginosa* is the same isolate used previously (Bérubé et al., 2010). CnT-BM.1 cell culture media and A, B, and C supplements (CnT-17.S) were purchased from CELLnTEC (Bern, Switzerland). Nuclear/cytoplasmic extract kit was purchased from Active Motif (Carlsbad, CA, USA, cat #40010).

### Cell culture

In this study we used two immortalized airway epithelial cell lines: one expressing the most common mutation found in CF, deletion of Phe508, termed UNC CF2, and referred to as CFTRdelF508 in this manuscript and another cell line, which expresses the wild type CFTR protein termed UNC N3 and referred to as wild type CFTR that were kindly provided by Dr. Scott Randell (The University of North Carolina at Chapel Hill, Chapel Hill, NC, USA; Fulcher et al., 2009). To enhance cell adherence, cells were seeded onto PureCol pre-coated plates (Advanced BioMatrix San Diego, California, USA). Cells were cultured in the medium CnT-17.S. All cells were maintained at 37°C in 5 % CO2, 100% humidity. The medium was changed every 48–72 h until cells reached 100% confluence. Cells were then synchronized with the medium CnT-BM.1 overnight and stimulated or infected as described.

### *Pseudomonas aeruginosa* infection

For acute bacterial infections, a clinical isolate of *Pseudomonas aeruginosa* (mucoid strain) was grown in LB Broth (Fisher Scientific) for 18 h at 37°C with shaking at 250 rpm and diluted to an optical density (OD) of 0.2 which corresponded to 3 × 10^9^ colony forming units (CFU) per mL. CF and non-CF airway epithelial cell lines were serum-starved overnight with CnT-BM.1 (CELLnTEC, Bern, Switzerland) without supplements or antibiotics. Bacteria were harvested by centrifugation, and re-suspended in CnT-BM.1. Cells were infected with 9 × 10^6^ CFU of *P. aeruginosa* per well and incubated for 3 h at 37°C. A 3 h infection was selected because it was the earliest time point where a significant induction of IL-33 could be detected.

### RNA extraction and real-time PCR

#### RNA extraction

RNA extraction was performed using TRIzol reagent (ThermoFischer Scientific). Briefly, after cell lysis with TRIzol, chloroform was added to separate the aqueous and by centrifugation at 12,000 × g at 4°C. Isopropanol (Fisher Scientific) as then added to precipitate RNA by centrifugation for 10 min at 12,000 × g at 4°C. The RNA pellet was washed with 75% ethanol and samples were then dried for 20 min at room temperature before being rehydrated in 5–10 μl sterile ribonuclease free water (Invitrogen). The diluted RNA quantified using a nano-drop system (Thermo Scientific).

#### Reverse transcription and quantitative real-time PCR

Potential DNA contamination from RNA samples was removed with DNAseI (Fermentas, Burlington, Ontario, Canada). Complementary DNA (cDNA) synthesis was then performed using Maxima Reverse Transcriptase (Fermentas). After addition of the mix containing reverse transcriptase, an RNAse inhibitor, random hexamer, and a solution with the 4 deoxynulceotide triphosphates, samples were incubated in a thermal cycler (BioRad My Cycler) as follows: 10 min at 25°C, 30 min at 50°C to achieve full polymerase activity and 5 min at 85°C to inactivate the enzyme.

For quantitative real-time PCR samples were assayed in Fast 96-well reaction plate (Applied Biosystems, Foster City, CA, USA) with each condition containing 100 ng of cDNA in a total volume of 2.5 μL sterile water with 0.3 μM of each forward and reverse primer (Integrated DNA Technologies, Coralville, IA), 5 μl iTAQ SYBR Green Supermix with Rox (BioRad) as well as 1.9 μl sterile water. Quantitative real-time PCR (qRT-PCR) was carried out in a Step-One-Plus machine (Applied Biosystems, Foster City, CA). Each condition was normalized to the housekeeping gene GAPDH. Relative fluorescence was interpreted as fold induction from cycle threshold values using the Pfaffl mathematical model.

Primer sequences are:

**Table:** 

**Gene**	**Forward primer (5′–3′)**	**Reverse primer (5′–3′)**
GAPDH	AGC AAT GCC TCC TGC ACC ACC	CCG GAG GGG CCA TCC ACA GTC
IL-33	CAA AGA AGT TTG CCC CAT GT	AAG GCA AAG CAC TCC ACA GT
IL-1α	ACC TCA CGG CTG CTG CAT TAC ATA	TGG TCT TCA TCT TGG GCA GTC AC
HMGB1	GGA GAT CCT AAG AAG CCG AGA	CAT GGT CTT CCA CCT CTC TGA
S100A9	AAA GAG CTG GTG CGA AAA GA	TCA GCT GCT TGT CTG CAT TT
IL-8	GTG CAG TTT TGC CAA GGA GT	CTC TGC ACC CAG TTT TCC TT

### Cell lysis and immunoblotting

Following stimulation, cells were lysed in 80 μL buffer containing 50 mM Tris-HCl pH 7.5, 1 mm EGTA, 1 mm EDTA, 1% (v/v) Triton x-100, 1 mm sodium orthovanadate, 5 mm sodium pyrophosphate, 50 mM sodium fluoride, 0.27 M sucrose, 5 mM sodium pyrophosphate decahydrate, one complete mini protease inhibitor mixture (Roche, Mannheim, Germany), and 2 mm DTT. Cell debris were separated from soluble proteins by centrifugation for 5 min at 4°C at 12,000 × g. 70 μL of supernatants were then added to 23 μL of loading buffer (0.24 mM Tris-HC, 8% SDS, 40% glycerol, and 36% distilled water) containing 1X TCEP (Thermo Scientific). A portion of the supernatant was retained for quantification using the Bradford method.

Quantified and normalized proteins were boiled for 5 min at 95°C. For each gel 30 μg of protein were deposited per well and separated by SDS-PAGE on a 10% Pro-pure Next Gel with Pro-Pure Running Buffer (Amresco, Solon, OH) using BioRad Powerpac HC (150 V/3.0 A/300W) at 150 V for 90 min. The proteins were then transferred from the gel to a nitrocellulose membrane using ice-cold 1X NuPAGE transfer buffer (Fisher Scientific), 10% methanol (v/v) for 35 min at 100 V. After blocking with 5% BSA, the membranes were incubated with primary antibodies for total ERK1/2 MAPK (1:500 dilution) and for phosphor-ERK1/2 MAPK (1:1000 dilution) overnight at 4°C. Antibodies were washed off using TBS with 0.05% Tween 20 (Sigma Aldrich) and secondary antibodies of goat anti-rabbit IgG DyLight™800 (Thermo Scientific, Cat #35571) and/or goat anti-mouse IgG DyLight™680 (Thermo Scientific, Cat #35518) were applied for 45 min in the dark at room temperature at a dilution of 1:15,000 in TBS 0.05% Tween. The membranes were washed as before and the signal was detected and quantified using a Licor Odyssey imaging system.

### Quantification of extracellular and intracellular IL-33 by ELISA

CFTRdelF508 cells were cultured in purecol-coated 6 well plates, grown to 100% confluence and synchronized with CnT-BM.1 as described in Section Cell Culture. Cells were then pre-incubated in the presence of different kinase inhibitors or DMSO (vehicle) and infected with *Pseudomonas aeruginosa* (MOI of 2) for 6 h. Supernatants were then harvested and stored at −80°C. Cytoplasmic proteins were obtained with the Active Motif nuclear/cytoplasmic extract kit. Briefly, following infection, cells were washed twice with ice-cold PBS containing phosphatase inhibitors, detached, and transferred to a pre-chilled microcentrifuge tube. Cells were then precipitated by centrifugation, supernatant was aspirated and the cell pellet was resuspended in 1X hypotonic buffer. After 15 min on ice, each sample was vortexed for 10 s at the highest setting. Cell debris were precipitated by centrifugation for 2 min at 13,000 × g at 4°C and supernatants containing cytoplasmic proteins were transferred to pre-chilled microcentrifuge tubes and stored at −80°C. IL-33 protein levels were measured by ELISA with the human IL-33 DuoSet Elisa kit (R&D systems cat#DY3625-05).

### Statistical analysis

Results were analyzed with GraphPad Prism software (version 5.0), *p*-values less than 0.05 were considered to be significant. Groups were compared by One-way ANOVA followed by Bonferroni post-test comparing control *vs*. infected or infected vs. the different inhibitors, ^*^p < 0.05, ^**^p < 0.01, and ^***^p < 0.001. Figures generated from this analysis are presented as means ± standard error of the mean (SEM) of three independent experiments.

## Results

### *P. aeruginosa* infection increases IL-33 expression in a CFTRdelF508 AEC line

**Figure 1:**
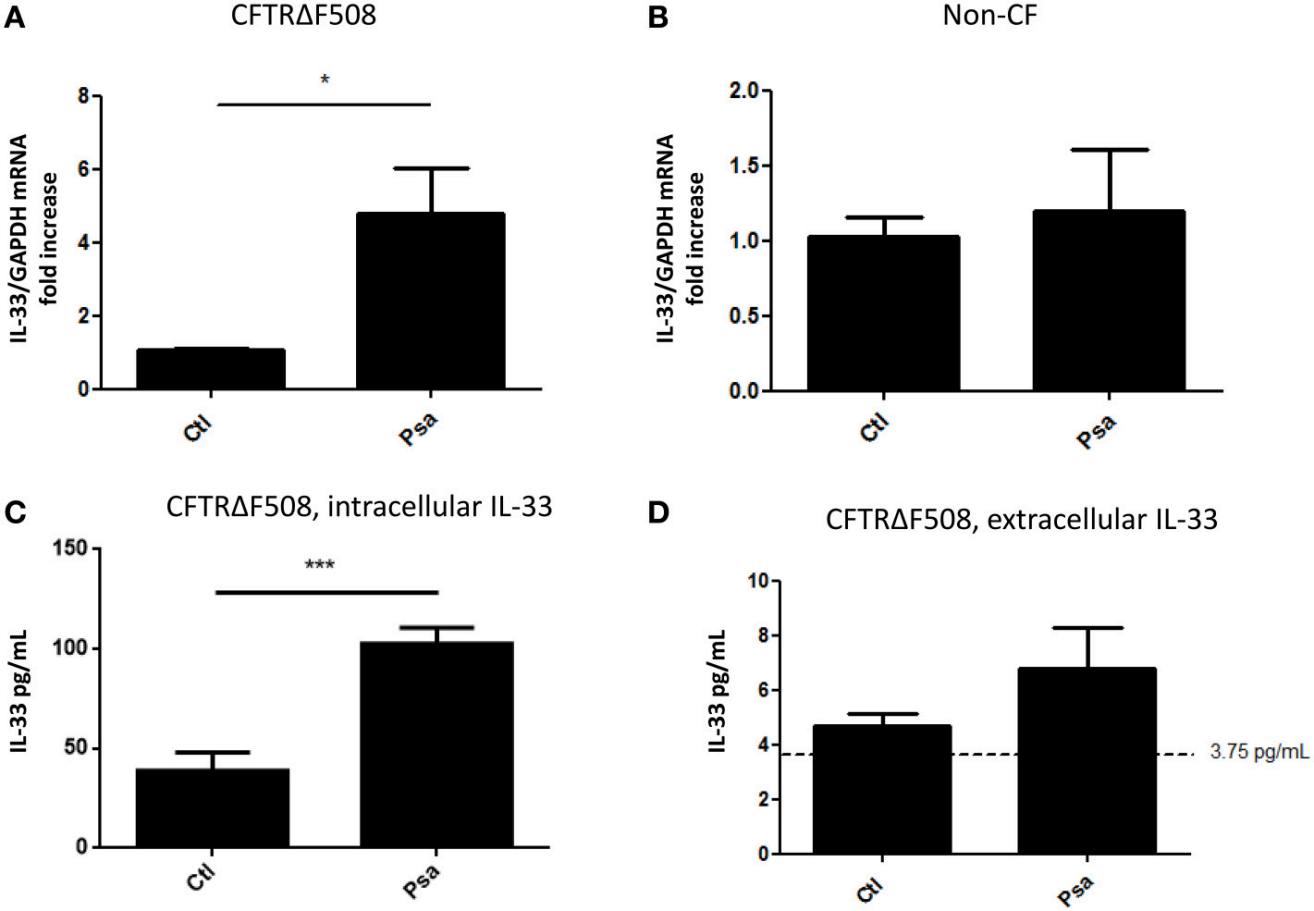
**Acute infection of a CFTRdelF508 cell line with ***Pseudomonas aeruginosa*** increases IL-33 expression. (A,B)** CF airway epithelial cells expressing the CFTRdelF508 mutation **(A)** and their wild-type counterparts **(B)** were infected with 9 × 10^6^ CFU of *P. aeruginosa* (MOI of 18) for 3 h and relative IL-33 gene expression was measured by qRT-PCR. **(C,D)** CFTRdelF508 cells were infected with *Pseudomonas aeruginosa* (MOI of 2) for 6 h. Supernatants were collected and frozen before the cells were lysed in a hypotonic solution as described in the methods section. IL-33 levels in the intracellular **(C)** and extracellular **(D)** fractions were quantified by ELISA. The dotted line at 3.75 pg/mL represents the limit of detection by ELISA. Results of three independent experiments were analyzed by one-tailed *t*-tests, ^*^p < 0.05, ^***^p < 0.001.

We have previously reported that diffusible material from a *P. aeruginosa* clinical isolate (PsaDM) leads to increased expression of IL-33 in CF airway epithelial cell lines (Roussel et al., 2013). PsaDM, which contains exoproducts derived from bacterial growth, mimics the chronic state of inflammation, where bacterial cells are not in direct contact with the epithelium (Worlitzsch et al., 2002). However, decline in lung function has been associated with episodes of pulmonary exacerbations (Sanders et al., 2010). Therefore, to mimic pulmonary exacerbations, we determined whether IL-33 was also upregulated during an acute bacterial infection. Similarly, to our previous results, infection with live *P. aeruginosa* led to an increase in IL-33 mRNA in a CFTRdelF508 AEC line but not in its wild type counterpart (Figures 1A,B). We then wanted to determine whether the increase of IL-33 mRNA led to an increase in protein expression. IL-33 is mainly localized in the nucleus of structural cells and is preferentially released upon necrotic cell death (Roussel et al., 2008; Cayrol and Girard, 2009). To determine if the acute infection with *P. aeruginosa* led to intracellular IL-33 accumulation vs. extracellular release, we measured IL-33 protein levels both in the intracellular and extracellular compartments. Interestingly, the increased mRNA expression was accompanied by increased detection of intracellular but not extracellular IL-33 following a 6 h infection of CFTRdelF508 AECs with *P. aeruginosa* (Figures 1C,D).

### CFTR correction and potentiation decrease IL-33 mRNA expression in a CFTRdelF508 AEC line

**Figure 2:**
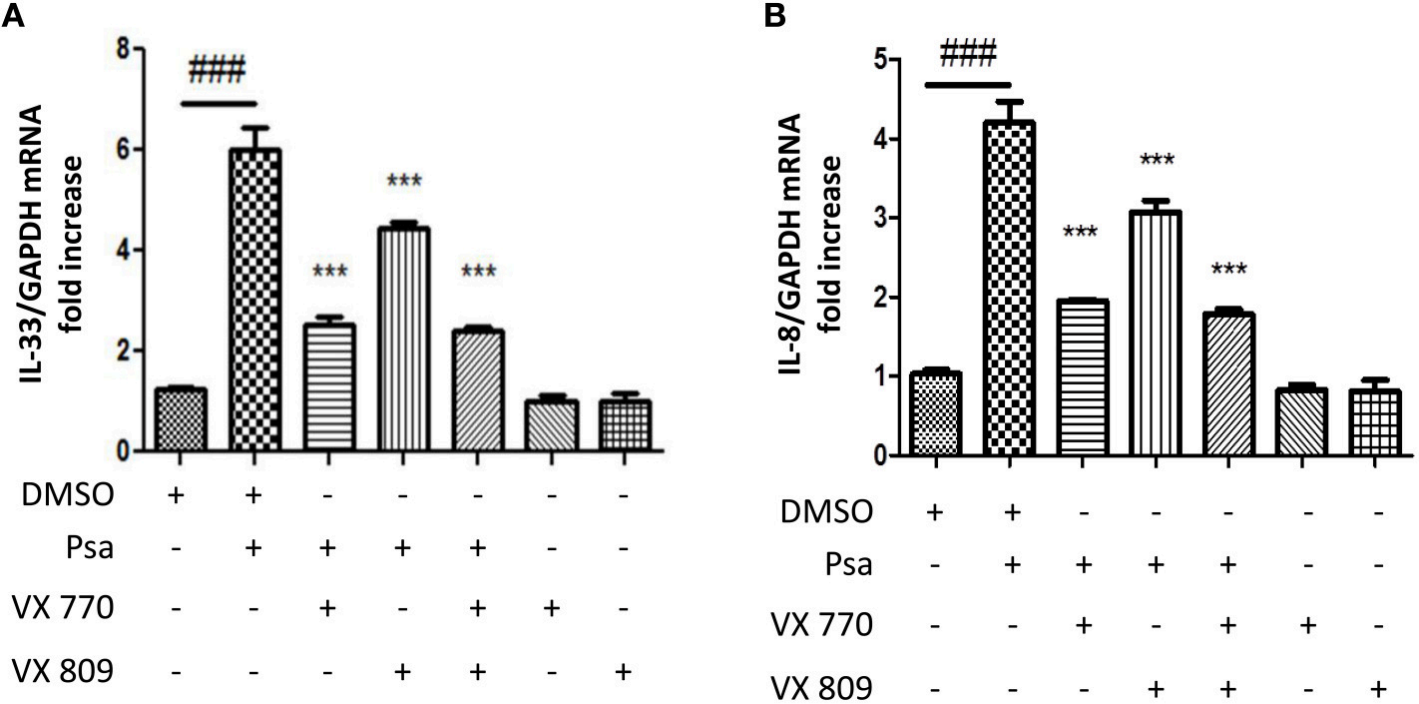
**IL-33 mRNA upregulation in response to infection is dependent on CFTR channel function in CFTRdelF508 airway epithelial cells**. **(A,B)** CFTRdelF508 cells were incubated in the presence of the CFTR corrector VX-770 (1 μM), the CFTR potentiator VX-809 (5 μM) or a combination of both for 24 h. Cells were then infected with *P. aeruginosa* (MOI of 18) for 3 h and IL-33 **(A)** and IL-8 expression **(B)** were measured by qRT-PCR. Results were analyzed by One-way ANOVA followed by Bonferroni post-test comparing control vs. infected (#), infected vs. the corrector or potentiator (^*^) and control vs. the corrector or potentiator alone. ^*###*^*p* < 0.001, ^***^p < 0.001.

Small molecule CFTR modulators are currently used as pharmacological strategies to correct CFTR defects. Compounds that revert processing and folding defects are termed correctors and those that increase channel gating and conductance are termed potentiators (Lubamba et al., 2012). We used this pharmacological approach to determine whether CFTR function was directly related to IL-33 expression. CFTR channel expression to the membrane was enhanced with the corrector VX-809 (Lumacaftor) and its channel activity was increased with the potentiator VX-770 (Ivacaftor) (Wainwright Claire, 2015). Neither, the corrector nor the potentiator alone affected baseline IL-33 expression (Figure 2A). Pre-treating CFTRdelF508 AECs with either compound, or in combination, decreased the *P. aeruginosa*-driven IL-33 (Figure 2A) and IL-8 (Figure 2B) mRNA expression. These results support the notion that the increase in IL-33 mRNA in CFTRdelF508 airway epithelial cells in response to infection is due to CFTR malfunction as it was previously shown for IL-8 (Veit et al., 2014).

### *P. aeruginosa*-driven IL-33 mRNA expression is dependent on TLR2 and TLR5

**Figure 3:**
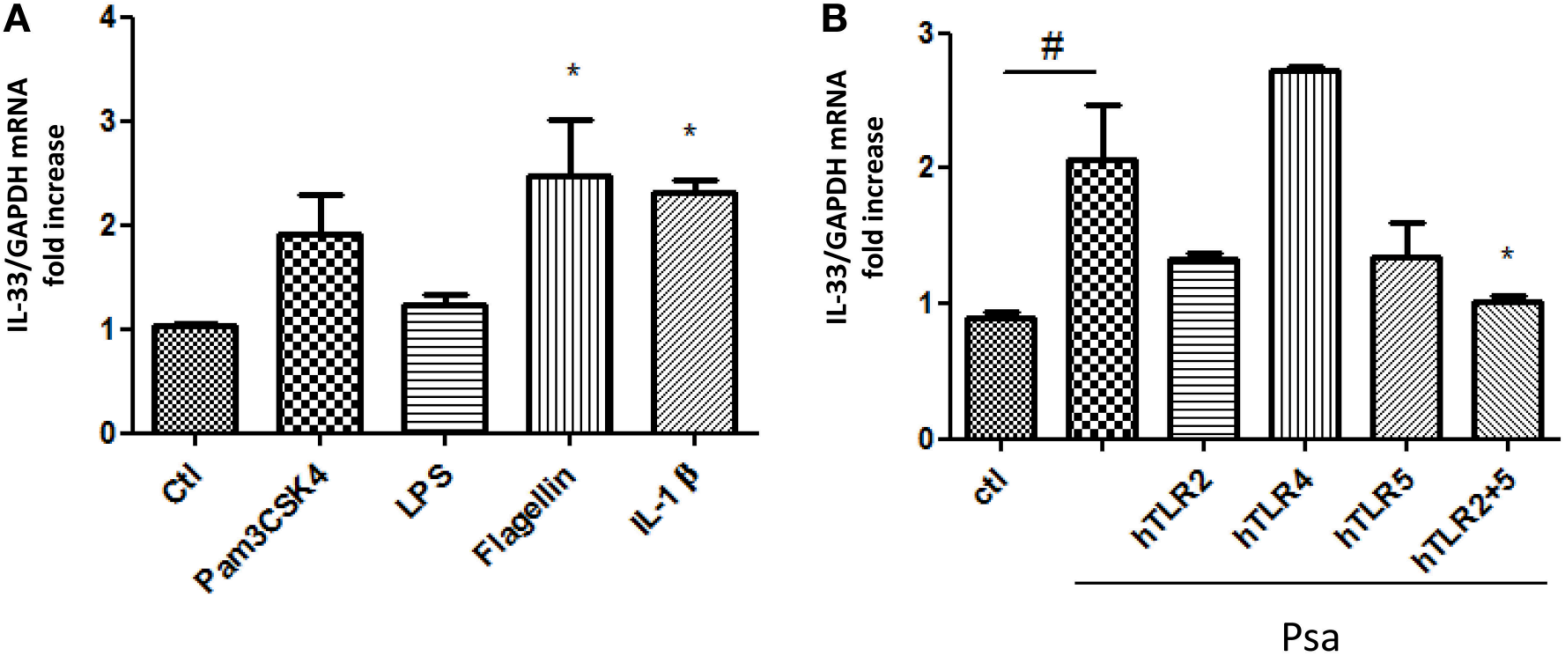
**IL-33 expression is regulated through TLR2 and TLR5-activated signaling following an acute infection with ***Pseudomonas aeruginosa*****. **(A)** CFTRdelF508 cells were stimulated with the following TLR receptor agonists for 6 h: Pam3CSK4 (TLR1/2), 1 μg/mL; LPS (TLR4), 1 μg/mL; Flagellin (TLR5), 400 ng/mL and IL-1 β, 100 ng/mL. IL-33 mRNA expression was measured by qPCR **(B)**. CFTRdelF508 cells were incubated with 20 μg/mL of the neutralizing antibodies against TLR2, TLR4, TLR5, and a combination of TLR2 and TLR5 for 30 min. Cells were then infected with 9 × 10^6^ CFU of Psa (MOI of 18) for 3 h, in the presence of the TLR neutralizing antibodies. IL-33 mRNA expression was measured by qPCR. Results from three independent experiments were analyzed by One-way ANOVA followed by Dunnett **(A)** or Bonferroni **(B)** post-tests, ^*^p < 0.05, ^#^*p* < 0.05.

In monocytes, TLR2 and TLR4 activation was shown to increase IL-33 expression (Nile et al., 2010). On the other hand, we have shown that TLR5 and TLR2 are the two main receptors activated by diffusible material from the clinical mucoid isolate of *P. aeruginosa* used to stimulate cytokine production by AECs (Beaudoin et al., 2012). To determine whether any of these receptors could lead to an increase in IL-33 expression, we stimulated CFTRdelF508 AECs with specific agonists for TLR2, TLR4, and TLR5. IL-1β was used as a positive control for IL-33 upregulation (Onda et al., 1999). A significant increase of IL-33 mRNA was seen when CFTRdelF508 AECs were exposed to the TLR5 agonist Flagellin and a trend to increase expression can be observed upon exposure to the TLR1/TLR2 agonist Pam3CSK4 (Figure 3A). To identify the receptor(s) involved in transducing *P. aeruginosa* signals, we prevented the activation of TLR2, TLR4, and TLR5 using neutralizing antibodies. Neutralization of both TLR2 and TLR5 blocked IL-33 mRNA upregulation in response to *P. aeruginosa* (Figure 3B). In accordance with low levels of TLR4 activation in AECs (Bérubé et al., 2009; Beaudoin et al., 2012), no major role has been found for this receptor, in contrast to monocytes/macrophages, where the TLR4 agonist LPS significantly increases IL-33 mRNA (Polumuri et al., 2012). We have previously reported poor responsiveness to LPS in other immortalized AECs (Bérubé et al., 2009; Beaudoin et al., 2013). This could be due to a low level of expression of TLR4 at the surface of AECs. Altogether, these results show that the expression of IL-33 in CFTRdelF508 AECs exposed to *P. aeruginosa* occurs via the activation of both TLR2 and TLR5.

### *P. aeruginosa*-driven IL-33 mRNA expression is dependent on the TAK1→IKKβ→TPL2→MKK1/MKK2 signaling pathway

**Figure 4:**
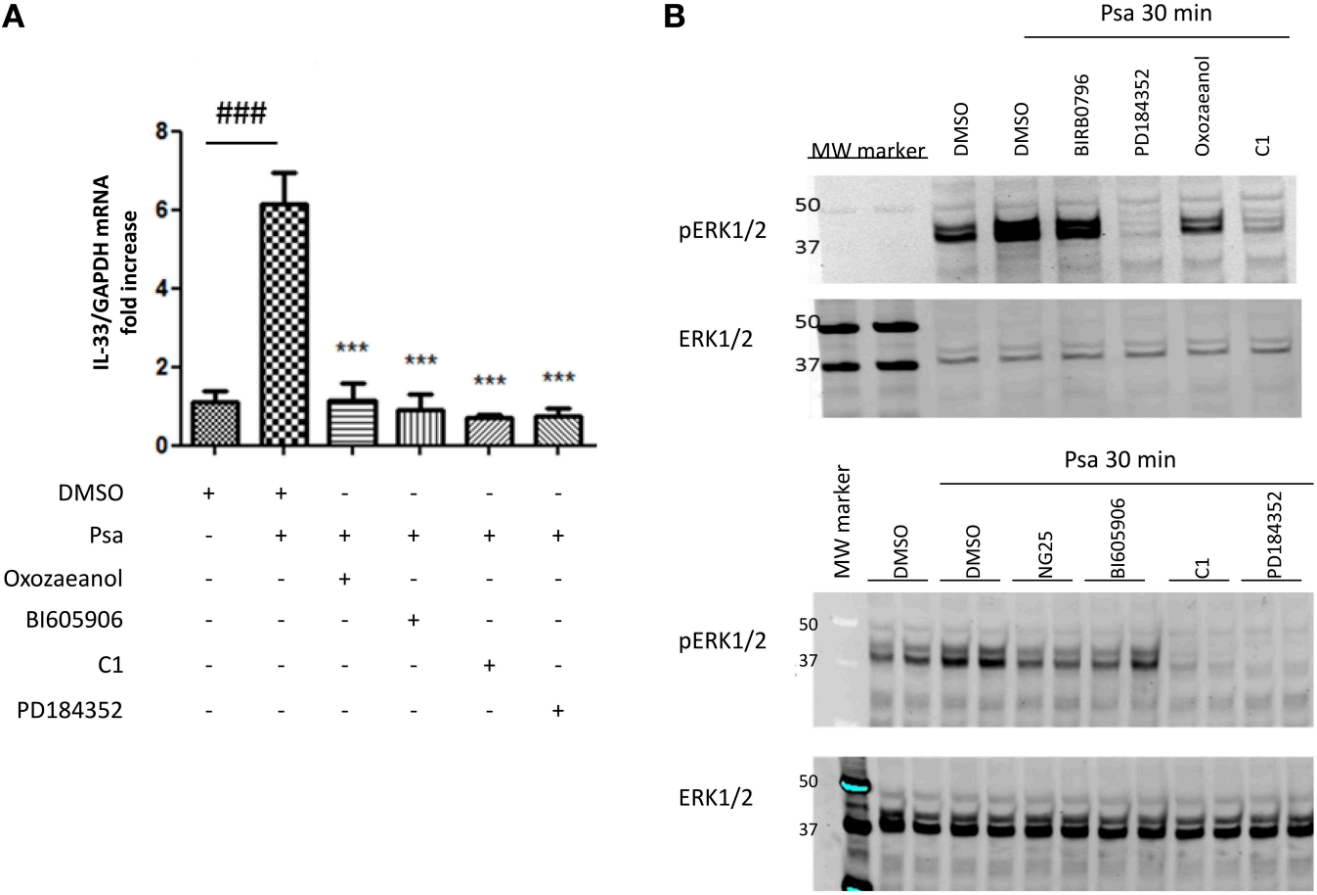
*****Pseudomonas aeruginosa***-driven IL-33 mRNA expression is dependent on the TAK1→IKKβ→TPL2→MKK1/MKK2 MAPK signaling pathway**. **(A,B)** CFTRdelF508 cells were pre-incubated for 1 h with DMSO (vehicle) or with one of the following inhibitors: PD184352, 2 μM; BI605906, 7.5 μM; C1, 2 μM, or 5Z-7-oxozeaenol, 250 nM. Then, cells were infected with 9 × 10^6^ CFU of *P. aeruginosa* (MOI of 18) for 3 h **(A)** or 30 min **(B)**. **(A)** IL-33 mRNA expression was analyzed by qRT-PCR. **(B)** CFTRdelF508 cells were pre-incubated with the p38 MAPK inhibitor BIRB0796, 0.1 μM, (added as a control), the MKK1/2 inhibitor PD184352, the TAK1 inhibitor 5Z-7-oxozeaenol and the TPL2 inhibitor C1. Cells were infected with *P. aeruginosa* and ERK1/ERK2 phosphorylation was measured by immuno-blotting. **(B)** In addition to the TPL2 and MKK1/2 inhibitors, *P. aeruginosa*-induced ERK1/2 MAPK phosphorylation was assessed in the presence of another TAK1 inhibitor NG25, 10 μM and the IKKβ inhibitor BI605906. Results from 4A were compared by One-way ANOVA followed by Bonferroni *post-hoc* test, comparing control vs. infected (#) or infected vs. the different inhibitors (^*^), ^***^ or ^*###*^*p* < 0.001.

The next step was to define the intracellular signaling pathways involved in controlling the expression of IL-33. TLR2 and TLR5 signal via the adaptor protein Myd88 as described in the introduction. To study the involvement of specific molecules activated downstream of Myd88, we used the following pharmacological inhibitors: we prevented TAK1 activity using 0.25 μM 5Z-7-oxozeaenol (Ninomiya-Tsuji et al., 2003), IKKβ, activity using 7.5 μM BI605906 (Clark et al., 2011), TPL2 activity using 2 μM C1 and MKK1/MKK2 activity using 2 μM PD184352 as previously reported (Martel et al., 2013). Pre-incubating CFTRdelF508 AECs with these protein kinase inhibitors showed that the activity of TAK1, IKKβ, TPL2, and MKK1/MKK2 was required for IL-33 expression in CFTRdelF508 AECs (Figure 4A). Accordingly, ERK1/ERK2 phosphorylation was prevented by inhibitors of TAK1, IKKβ, TPL2, and MKK1/MKK2 but only barely by the p38 MAPK inhibitor BIRB0796 (Figure 4B).

### *P. aeruginosa* infection increases IL-1α and S100A9 mRNA expression but not that of HMGB1

**Figure 5:**
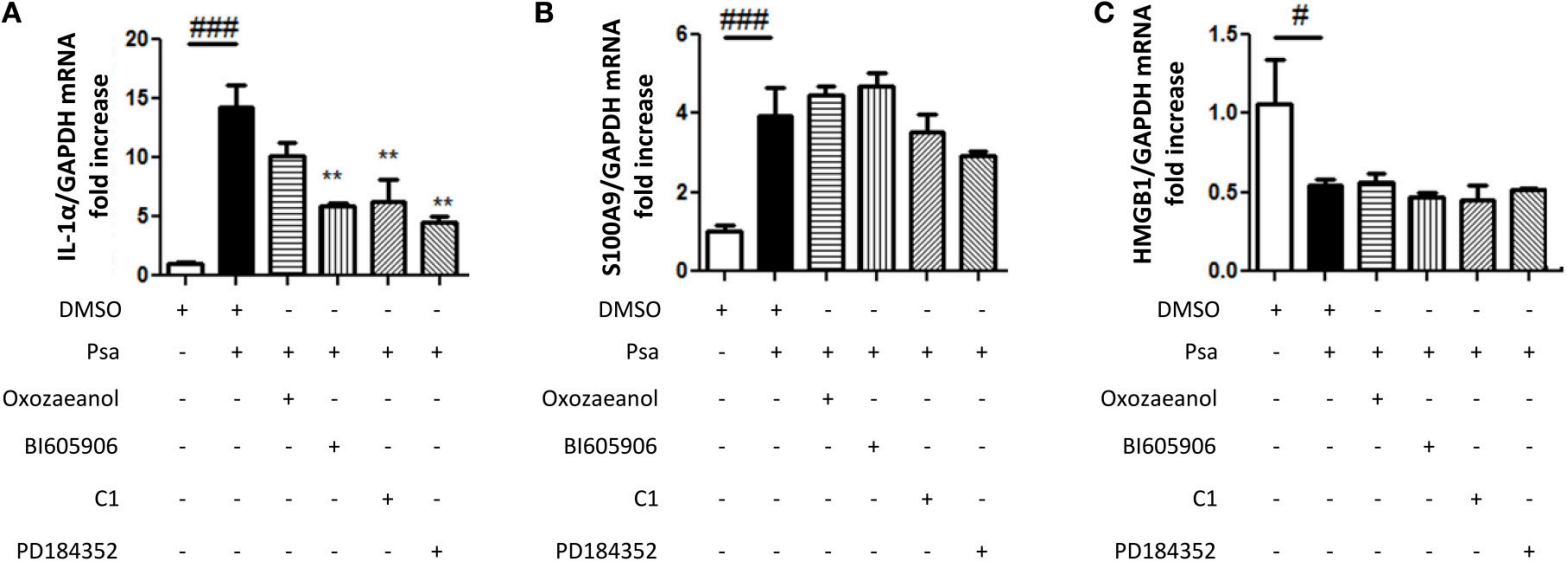
*****Pseudomonas aeruginosa*** infection increases IL-1α and S100A9 mRNA expression but not that of HMGB1**. CFTRDELF508 airway epithelial cells were incubated in the presence of different kinase inhibitors as described in **(A)** and then infected with *P. aeruginosa* (MOI of 18) for 3 h. IL-1α **(A)**, S100A9 **(B)**, and HMGB1 **(C)** gene expression was measured by qRT-PCR. Results were analyzed by One-way ANOVA followed by Bonferroni post-test comparing control vs. infected (#) or infected vs. the different inhibitors (^*^), ^#^*p* < 0.05, ^**^p < 0.01, and ^*###*^*p* < 0.001.

IL-33 is a member of the IL-1 family, functionally related to IL-1α, since both are proposed to act as alarmins. *P. aeruginosa* infection of CF AECs also increases IL-1α mRNA expression (Figure 5A). To determine if this was specific to the IL-1 family, we also investigated the alarmins HMGB1 and S100A9. HMGB1 like IL-1α is found in CF sputa (Rowe et al., 2008; Colombo et al., 2011), whereas S100A9 is a part of Calprotectin, a complex described as the Cystic Fibrosis protein in 1973 (Wilson et al., 1973). Whereas *P. aeruginosa* infection of CF AECs led to increased S100A9 expression (Figure 5B), no increase was detected for HMGB1 (Figure 5C). If anything, lower levels were detected following infection (Figure 5C). IL-1α expression was also regulated by the TAK1→IKKβ→TPL2→MKK1/MKK2 cascade (Figure 5A), whereas none of the protein kinase inhibitors tested decreased the *P. aeruginosa*-driven S100A9 expression (Figure 5B).

## Discussion

**Figure 6:**
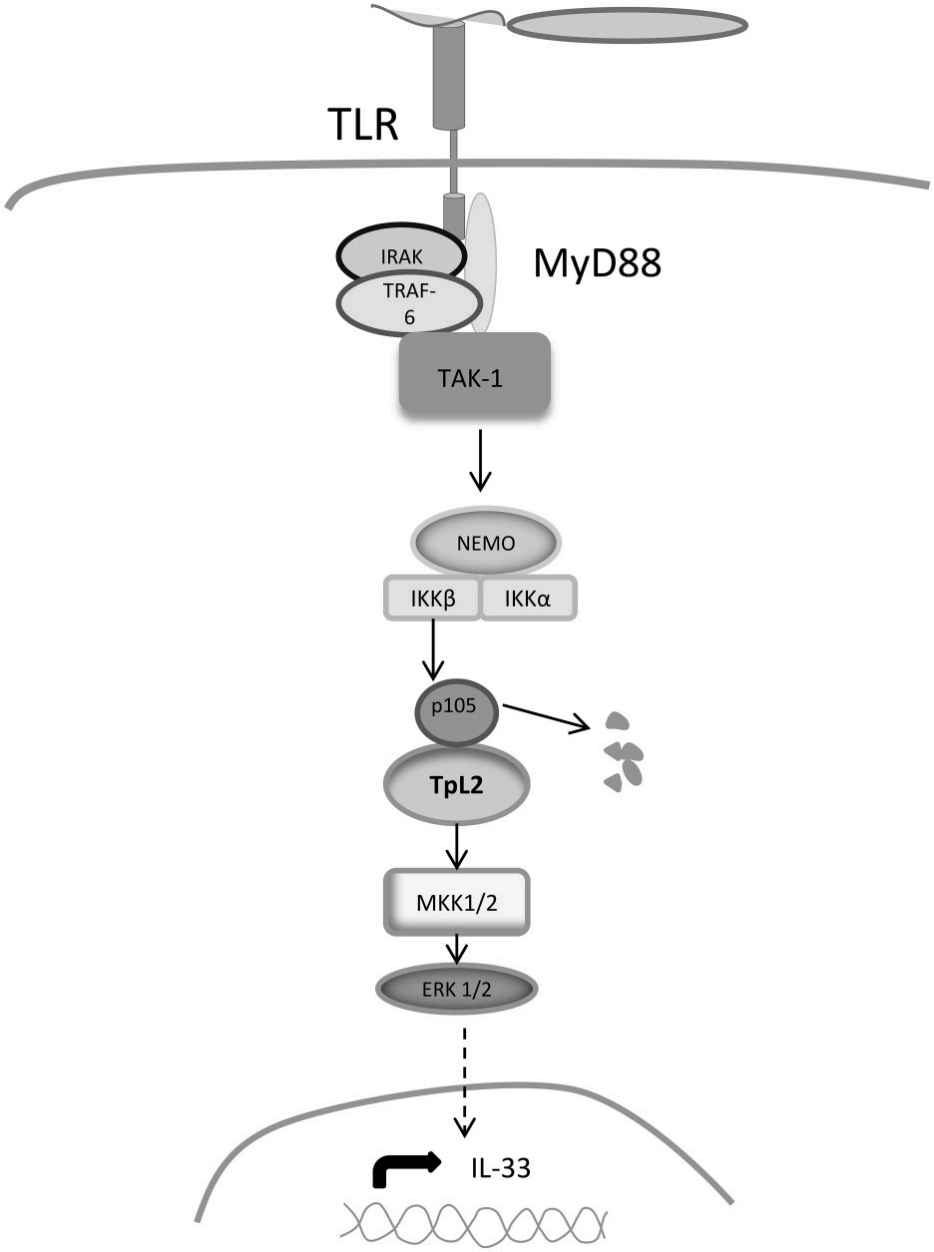
**Signaling mechanisms regulating IL-33 expression**. Binding of bacterial PAMPs to TLR2 and TLR5 leads to the recruitment of adaptor molecules that activate TAK1. TAK1 phosphorylates IKKβ, which in turn phosphorylates the NFκ B subunit p105, leading to its dissociation from TPL2. TPL2 is then released and is able to phosphorylate MKK1/MKK2, which activate ERK1/ERK2 MAPKs resulting in the nuclear translocation ERK1/ERK2 and in the activation of transcription factors that increase IL-33 expression.

In this manuscript we show that *P. aeruginosa* increases the expression of IL-33 in a CFTRdelF508 airway epithelial cell line via TLR2 and TLR5-mediated activation of the TAK1→IKKβ→TPL2→MKK1/MKK2→ERK1/2 signaling cascade (Figure 6).

In a previous study we had reported the presence of IL-33 in the epithelium of CF lungs and increased expression only in CF AEC lines when challenged with *P. aeruginosa* diffusible material (PsaDM) (Roussel et al., 2013), a challenge mimicking the chronic inflammatory state. Interestingly, the presence of IL-33 in Broncho-alveolar lavage fluid negatively correlated with lung function in CF patients (Tiringer et al., 2014). Following pulmonary exacerbations, CF patients undergo a net decline in lung function (Sanders et al., 2010). Some studies have attributed these episodes to the release of planktonic bacteria from the biofilms colonizing CF lungs leading to acute episodes of infection (Chattoraj et al., 2011). We report here that acutely infecting a CFTRdelF508 AEC line with a live clinical isolate of *P. aeruginosa* also led to IL-33 upregulation. As reported for PsaDM (Roussel et al., 2013), this increased expression of IL-33 occurs preferentially in the absence of functional CFTR, the anion channel defective in CF. This is further supported by decreased IL-33 expression when CFTRdelF508 AECs are pre-treated with a CFTR corrector (VX-809) and/or potentiator (VX-770) before infection. Accordingly, IL-8 levels were also decreased by these pre-treatments, further supporting the link between CFTR malfunction and hyper-activation of the inflammatory response (Veit et al., 2014). However, we cannot completely rule out that VX-809 and VX-770 act through other CFTR-independent mechanisms to regulate inflammatory gene expression.

The molecular mechanisms responsible for this response in CF AECs are unknown. Our results have shown that TLR-mediated signaling regulates IL-33 expression in the context of infection. However, TLR-signaling is activated in both non-CF and CF AEC lines by PsaDM or live *P. aeruginosa* (Bérubé et al., 2010; Beaudoin et al., 2012, 2013). One possibility is the different levels of activation of TLR-signaling between non-CF and CF AECs. We have shown that CF AECs are hyper sensitive to a number of insults resulting in MAPK hyper-activation (Bérubé et al., 2010). Therefore, there may exist a threshold level required for IL-33 expression that is not reached in non-CF AECs. Another possibility is the activity of a parallel pathway that is permissive for IL-33 expression, which is not activated in non-CF AECs, preventing IL-33 expression. Redox balance seems to be impaired in CF AECs (Bérubé et al., 2010; Duranton et al., 2012), favoring a higher oxidant burden, which could activate such a parallel pathway. Interestingly, in astrocytes, the presence of ROS is associated with IL33 expression (Khan et al., 2014). Further, investigations are required to elicit the mechanisms linking the absence of CFTR to IL-33 expression.

Our results using pharmacological inhibitors have identified a key pathway required for IL-33 expression: the TAK1→IKKβ→TPL2→MKK1/MKK2 cascade leading to ERK1/ERK2 activation. Naturally, we cannot formally exclude that some of these inhibitors have acted through other protein kinases that they target “non-specifically.” This is the first time that activation of the protein kinases TAK1, IKKβ, and TPL2 is linked to the expression of the pro-inflammatory cytokine IL-33, illustrating further the importance of these protein kinases to the regulation of inflammation in the context of acute infection (Dumitru et al., 2000; Martel and Rousseau, 2014). ERK1/ERK2 role in IL-33 expression has previously been shown in myofibroblasts stimulated with TNFα or IL-1β (Kobori et al., 2010). Our data further strengthen the importance of the activation of ERK1/ERK2 for expression of IL-33 in response to inflammatory stimuli. It is well known that TAK1 signaling can also result in the activation of p38 and JNK MAPKs. We have not investigated these other TAK1-activated pathways in this study.

TAK1 and IKKβ activity are not only required for TPL2 activation but also lead to the activation of NFkB signaling. We have not assessed directly whether NFkB is needed for IL-33 expression in our CF AECs exposed to *P. aeruginosa*, but in TNFα or IL-1β-stimulated myofibroblasts expressing a dominant-negative form of IkB, IL-33 expression was impaired (Kobori et al., 2010).

The expression of the related cytokine IL-1α, appears to be very similarly regulated, however that of the unrelated alarmin S100A9 occurs in a TAK1-indepent manner. Despite the high level of transcripts, we did not detect increases in the expression of HMGB1 by epithelial cells stimulated with *P. aeruginosa*. The presence of HMGB1 in CF sputa (Rowe et al., 2008) may be due to other cells such as macrophages (Jiang et al., 2007), which are an abundant source of this alarmin, or neutrophils, which are present in great numbers in CF sputum.

Overall, our results have defined a signaling cascade leading to an increase of IL-33 expression in CF AECs in response to an acute infection. We postulate that this increased expression is linked to the diseased state of CF lungs and reducing IL-33 expression may have benefits in limiting inflammation in CF lungs. In that regard, targeting the protein kinases in the TAK1→IKKβ→TPL2→MKK1/MKK2 signaling pathway represents an attractive option.

## Author contributions

RF and SR have made substantial contributions to the conception, design, acquisition, analysis, and interpretation of data for the work. RF and SR have drafted the work and revised it critically for intellectual content. RF and SR have approved the final version to be published and agreed to be accountable for all aspects of the work.

## Funding

We acknowledge the financial support of Cystic Fibrosis Canada and the Canadian Institute of Health Research (MOP#123496). The Meakins-Christie Laboratories – MUHC-RI, are supported by a Centre grant from Les Fonds de Recherche du Québec- Santé (FRQ-S). SR acknowledges a salary award from the FRSQ. RF acknowledges a salary award from the Mexican program CONACYT.

### Conflict of interest statement

The authors declare that the research was conducted in the absence of any commercial or financial relationships that could be construed as a potential conflict of interest.

## Abbreviations

AECs: airway epithelial cells
CF: cystic fibrosis
CFTR: cystic fibrosis transmembrane conductance regulator
DAMPs: damage associated molecular patterns
ERK: extracellular signal-regulated kinase
HMGB1: high mobility group box 1
IKKβ: I-kappa-B kinase beta
IκB: inhibitor of kappa light polypeptide gene enhancer in B-cells
IL: interleukin
LPS: lipopolysaccharide
MAPK: mitogen-activated protein kinase
MKK: mitogen-activated protein kinase kinase
Myd88: myeloid differentiation primary response 88
NF-kB: nuclear factor-kB
NFkB1-p105: nuclear factor of kappa light polypeptide gene enhancer in B-cells 1
PRR: pattern recognition receptors
PsaDM: *Pseudomonas aeruginosa* diffusible material
TAK1: TGF-beta activated kinase 1
TLR: toll-like receptor
TNF: tumor necrosis factor
TPL2: tumor progression locus 2
TRAF6: TNF receptor-associated factor 6.
